# The Effects of Action Observation Speed on Motor Function in Patients with Chronic Low Back Pain: From Observation to Execution

**DOI:** 10.3390/brainsci15010031

**Published:** 2024-12-30

**Authors:** Mónica Grande-Alonso, Manuel Estradera-Bel, Carlos Forner-Álvarez, Ferran Cuenca-Martínez, Celia Vidal-Quevedo, Alba Paris-Alemany, Roy La Touche

**Affiliations:** 1 Departamento de Cirugía, Ciencias Médicas y Sociales, Facultad de Medicina, Universidad de Alcalá, 28871 Alcalá de Henares, Spain; monica.grande@uah.es; 2 Grupo de Investigación Clínico-Docente sobre Ciencias de la Rehabilitación (INDOCLIN), Centro Superior de Estudios Universitarios La Salle, 28023 Madrid, Spain; 3 Unidad de Trastornos Musculoesqueléticos, Instituto de Rehabilitación Funcional La Salle, Centro Superior Estudios Universitarios La Salle, 28023 Madrid, Spain; 4 Faculty of Physiotherapy, University of Valencia, c/Gascó Oliag n°5, 46010 Valencia, Spain; 5 Department of Physiotherapy, University of Valencia, c/Gascó Oliag n°5, 46010 Valencia, Spain; 6 Servicio de Rehabilitación, Instituto de Investigación Sanitaria Fundación Jiménez Diaz (IIS-FJD, UAM), Hospital Universitario Rey Juan Carlos, 28040 Madrid, Spain; 7 Departamento de Radiología, Rehabilitación y Fisioterapia, Facultad de Enfermería, Fisioterapia y Podología, Universidad Complutense de Madrid, 28040 Madrid, Spain; 8 Motion in Brains Research Group, Institute of Neuroscience and Sciences of the Movement (INCIMOV), Centro Superior de Estudios Universitarios (CSEU) La Salle, Universidad Autónoma de Madrid, 28023 Madrid, Spain; 9 Instituto de Neurociencia y Dolor Craneofacial (INDCRAN), 28003 Madrid, Spain; 10 Centro Superior de Estudios Universitarios (CSEU) La Salle, Universidad Autónoma de Madrid, 28023 Madrid, Spain

**Keywords:** chronic low back pain, action observation, Timed Up and Go test, lumbar flexion

## Abstract

Objective: The objective of this study was to examine the effect of observing actions at different speeds on the speed of motor task performance in subsequent actions. Methods: Sixty individuals, divided equally between those with non-specific chronic low back pain (NSCLBP) and asymptomatic subjects, were enrolled. Participants were further split into subgroups to observe lumbar flexion and Timed Up and Go (TUG) test actions at either a slow or fast pace, following a randomized assignment. For post-video observation, participants replicated the observed actions three times without specific performance instructions, allowing for the assessment of their execution speed. Results: The analysis revealed that individuals observing actions at a faster pace executed the subsequent motor tasks significantly quicker than their counterparts who viewed the same actions at a slower speed. This was consistent across both NSCLBP sufferers and asymptomatic subjects, indicating that the action observation (AO) speed directly influenced the execution speeds of lumbar flexion and TUG test movements. Conclusions: The findings demonstrate that AO speed significantly affects the pace of motor execution, irrespective of NSCLBP presence. This underscores the potential of utilizing varied AO speeds as a strategic component in clinical practice, particularly for enhancing motor planning and execution in physical therapy settings. The study highlights the importance of incorporating AO speed variations into therapeutic interventions for improving patient outcomes in motor task performance.

## 1. Introduction

Low back pain (LBP) is the most prevalent musculoskeletal problem, with the greatest socioeconomic impact [1,2,3,4]. It is the main cause of work absenteeism and ranks third in the classification of diseases that generate the most disability, behind ischemic heart disease and chronic obstructive pulmonary disease [5]. Moreover, in some countries, such as Spain, low back pain is the second most frequent reason for medical consultation [6]. According to different classifications of LBP, the most prevalent situation, accounting for 85–90% of this disorder, is the presentation of nonspecific chronic LBP (NSCLBP). NSCLBP is defined as “tension, pain, and/or stiffness in the lumbar region for which no specific cause of pain can be identified”, and lasts for more than 3 months [7,8].

From a physical therapy point of view, the treatment of chronic musculoskeletal pain should be multimodal based on therapeutic exercise, manual therapy and therapeutic education, including the mental representation of movement techniques such as action observation (AO), which has shown positive results in recent years in reducing pain intensity [9]. The effect of AO is primarily due to the activation of mirror neurons when observing motor patterns [10]. When a human being observes an exercise, similar areas are activated to those activated when performing the same exercise [11,12,13]. In this line, previous studies show that patients with NSCLBP may present changes in motor learning, as it has been observed that they present an alteration in the activity of the primary motor area, directly affecting muscle function and coordination [14,15]. According to the available evidence, it is known that activating areas of the brain similar to those activated during performance, as it happens in AO, may influence movement planning and therefore motor performance [16]. Some studies show that observing actions leads to a significant improvement in variables such as strength, range of motion, static balance or walking speed [17,18,19]. Furthermore, it was found that AO can also influence factors such as fear that can have a direct impact on motor variables [20,21,22]. It is hypothesized that these improvements may be determined by the influence of the mirror neuron system found in the primary motor cortex, the premotor cortex, and the supplementary motor area [20,21].

Although aspects of AO such as its neurophysiology or its effects individually or in combination with other interventions have been of great scientific interest, little has been studied on the effect of modifying its parameters, such as the speed of AO reproduction. Manzone and Tremblay found that a change in the AO reproduction rate could produce effects on the processing of the movement and thus influence both its execution and characteristics [23]. However, for patients with NSCLBP, there is a lack of evidence regarding the modification of AO speed and its effects on movement characteristics. Regarding the movement characteristics, the speed of execution is one of the most interesting for several reasons. Firstly, in some pathologies, it is recommended to optimize the temporal variables of movements, among which is the speed of execution, as it was seen that this produces appropriate task-specific feedback which can cause beneficial effects on the activity of the motor cortex [24,25]. In both stroke and NSCLBP, there is usually an alteration of primary motor area activity [14,15,26], and in both, it has been shown that AO can produce effects [27,28]. Due to this relationship, it would be interesting to consider the possibility that, as in stroke, a higher speed of execution in NSCLBP may produce changes in the alteration of motor activity and therefore also have an impact on the impairments produced by the alteration. Secondly, at the clinical level, a higher speed of movement execution can be crucial in functional tests in which speed plays a fundamental role in the development of the test, and of which the results are associated with an improvement in the patient’s general condition. For example, better results in the Timed Up and Go (TUG) are associated with a lower risk of falling [29], while better results in the six-minute walking test or the two-minute step test indicate a higher exercise capacity of the patient [30,31]. Moreover, this is of great importance as it has been observed that a lower risk of falling and a greater exercise capacity value are associated with a better quality of life and a longer life expectancy [32,33]. With this background, we believe it is necessary to test whether a higher AO playback speed has a significant effect on movement characteristics, with special consideration of the speed of execution.

Based on what has been described above, it is hypothesized that the higher the speed of playback of the observed action, the higher the speed of execution of the action. Therefore, the main objective of the study was to assess whether the visualization of an action at a specific speed determines the subsequent execution rate.

## 2. Materials and Methods

### 2.1. Study Design

A cross-sectional observational study was conducted on patients with NSCLBP. The study protocol follows the Consolidated Standards of Reporting Trials (STROBE) statement [34]. The procedure of this study was approved by the ethics committee of the Higher Center for University Studies La Salle (CSEULS-PI-041/2021) and is in accordance with the Declaration of Helsinki. After receiving detailed information about the study, patients provided written informed consent. A total of 60 participants were recruited, including 30 patients with NSCLBP and 30 asymptomatic subjects. Each group was divided into two subgroups of 15 individuals, one being the fast-speed group and the other the slow-speed group. In total, four groups were formed.

Patients with NSCLBP were recruited from IRF La Salle, Hospital del Escorial, Podalba Clinic, and Ricondo Physiotherapy Clinic. The sample of asymptomatic subjects was recruited from the local community via telephone or verbal communication. Participants were recruited between December 2021 and July 2022. The inclusion criteria for the symptomatic group were (a) subjects with NSCLBP of at least 3 months in duration; (b) males and females aged between 18 and 65 years; (c) NSCLBP with a frequency of at least 10 days per month; and (d) presenting a pain intensity on the visual analog scale (VAS) of at least 30/100. The inclusion criteria for asymptomatic subjects were (a) subjects not suffering from LBP at the time of measurement; (b) males and females aged between 18 and 65 years; (c) not presenting pain at the time of evaluation; and (d) participants without systemic, neurological, cognitive, or psychological diseases. The exclusion criteria were (a) comorbidities such as neurological signs (lower limb weakness), rheumatoid systemic diseases (including fibromyalgia), or central nervous system diseases; (b) the presence of diagnosed psychiatric illness or severe cognitive impairment; (c) illiteracy; (d) difficulties in comprehension and communication; (e) insufficient knowledge of Spanish; and (f) participants studying for a degree in physiotherapy or occupational therapy.

### 2.2. Procedure

The procedure began with the recruitment of participants who met the inclusion and exclusion criteria. Once recruited, participants were provided with a brief overview of the study protocol and were asked to sign a written informed consent form. Next, participants underwent a series of assessments and measurements. This included demographic data collection such as age, gender, body mass index, education level, and occupation. Additionally, participants reported their current medication use and pain levels. After completing this part of the study, the patients watched two videos. It is significant to note that all the videos were made from an allocentric perspective in which the study subject viewed a third person in full body. The video was made by a young, male, adult non-study subject. It was recorded in a frontal plane. The visualization was performed on a 12″ screen of a Surface Pro 7.

The first video showed a person performing the lumbar flexion gesture ten times at a so called “normal” angular velocity of 45°/s. Then, without giving instructions to the patient, they were asked to perform the gesture three times.

In the second video, they watched a person perform the stand-up-and-go gesture ten times. Afterward, they were asked to repeat the gesture without instruction. Depending on which group they were assigned to, participants watched the gestures at different speeds. One group of pain patients and one group of asymptomatic individuals watched the videos at a faster speed, while the other group of pain patients and the other group of asymptomatic individuals watched them at a slower speed. The video with the slowest speed of the lumbar flexion gesture had an angular velocity of 14°/s and a range of motion of 37°. The video with the fastest speed had an angular velocity of 77°/s and a range of motion of 38°. In the case of the TUG, the video with the slower speed had a duration of 17.33 s, a vertical acceleration to stand up of 0.89 m/s^2^, and a vertical acceleration to sit down of 1.96 m/s^2^. The video with the faster speed had a total time of 4.98 s, with a vertical acceleration to stand up of 5.15 m/s^2^ and a vertical acceleration to sit down of 2.84 m/s^2^.

### 2.3. Outcome Measures

**Figure 1:**
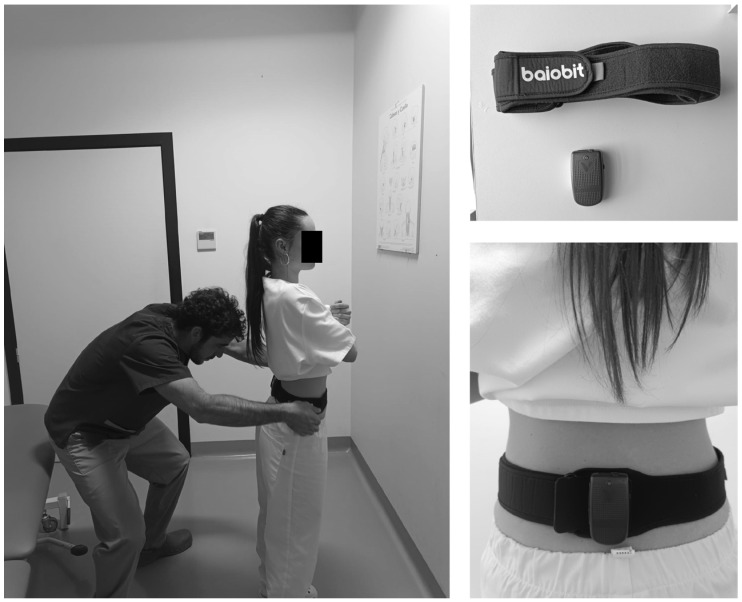
Images of the Baiobit device.

All variables were measured with the Baiobit^®^ device which is a system of the Rivelo group (Garbagnate Milanese, Italy) and BTS bioengineering group (Garbagnate Milanese, Italy) [35] (Figure 1). It is a portable and wireless device composed of accelerometers, a gyroscope, and a magnetometer. Acceleration-related data were transmitted via Bluetooth to a computer. It utilizes the Rivelo processing system [35]. Previous studies indicate that it is a valid measurement tool with an intraclass coefficient correlation ranging from 0.10 to 0.98, depending on the measured variable [36].

### 2.4. Primary Outcome

#### Lumbar Flexion Velocity

Lumbar flexion velocity refers to the speed at which the angular movement of the joints occurs, measured in degrees per second (°/s). It has been measured in the flexion gesture.

### 2.5. Secondary Outcome (Timed Up and Go)

#### 2.5.1. Total Time

Total time indicates the total time it takes to complete a specific movement, measured in seconds (s). It has been measured in the Timed Up and Go.

#### 2.5.2. Sit-Up Acceleration Phase

The sit-up acceleration phase is the rate of change of vertical velocity during the process of standing up, measured in meters per second squared (m/s^2^).

#### 2.5.3. Sit-Down Acceleration Phase

The sit-down acceleration phase refers to the rate of change of vertical velocity during the process of sitting down, also measured in meters per second squared (m/s^2^).

### 2.6. Sample Size

A pilot study determined the effect size between the AS and the NSCLBP using the main variable. The pilot study included a total of 5 participants from each group (4 groups) and an effect size f of 0.46. The sample size was calculated with the program G*Power 3.1.7 for Windows (G*Power© by the University of Düsseldorf, Düsseldorf, Germany) [37]. It was calculated using the between-group and within-group repeated measures ANOVA test using an α error level of 0.05, a statistical power of 80% (1-β probability of error), and an effect size of 0.46. This analysis generated a sample size of 15 participants per group.

### 2.7. Data Analysis

The statistical data analysis was performed using statistical SPSS software version 25.0 (SPSS Inc., Chicago, IL, USA). The normality of the variables was evaluated by the Shapiro–Wilk test. Descriptive statistics were used to summarize the data for continuous variables and are presented as the mean ± standard deviation, with a 95% confidence interval. The categorical variables are presented as absolute (number) and relative frequencies (percentage). A two-way repeated measures analysis of variance (ANOVA) was conducted to study the effect of the between-participant “intervention group” factor in each of the four categories (NSLBP (slow and fast AO) and asymptomatic subjects (slow and fast AO)) and the within-participant “time” factor, also in each of the two categories (pre-, and post-intervention) of all the dependent variables. The partial eta squared (η^2^) was calculated as a measure of effect size (strength of association) for each main effect and interaction in the ANOVAs, with 0.01–0.059 representing a small effect, 0.06–0.139 a medium effect, and >0.14 a large effect [38]. A post hoc analysis with Bonferroni correction was performed in the case of significant ANOVA findings for multiple comparisons between variables. The effect sizes (d) were calculated according to Cohen’s method, in which the magnitude of the effect was classified as small (0.20–0.49), moderate (0.50–0.79), or large (0.8) [39]. Finally, an accuracy analysis was performed by comparing the observed video velocity values with respect to the mean values obtained by each group, both in the lumbar flexion test and in the TUG time by the means of a one-sample *t*-test. The α level was set at 0.05 for all tests.

## 3. Results

**Figure 2:**
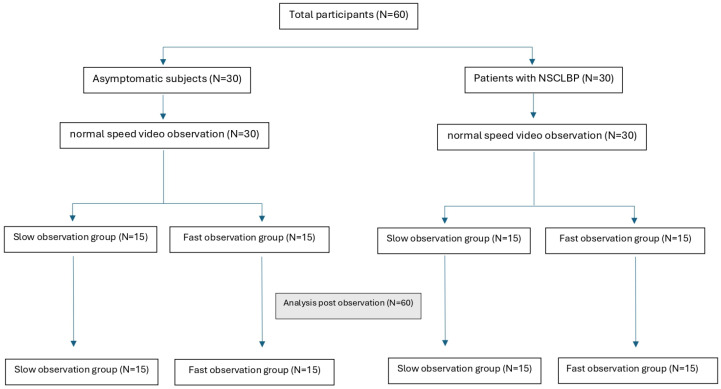
Diagram flow chart.

A total of 60 participants (30 patients with NSCLBP and 30 AS) were randomly divided to four subgroups (15 patients with NSCLBP in the fast observation group, 15 patients with NSCLBP in the slow observation group, 15 AS in the fast observation group and finally 15 AS in the slow observation group) (Figure 2). There were no adverse events reported in either group. Table 1 shows the sociodemographic data and self-reported variables.

### 3.1. Lumbar Flexion Velocity

**Figure 3:**
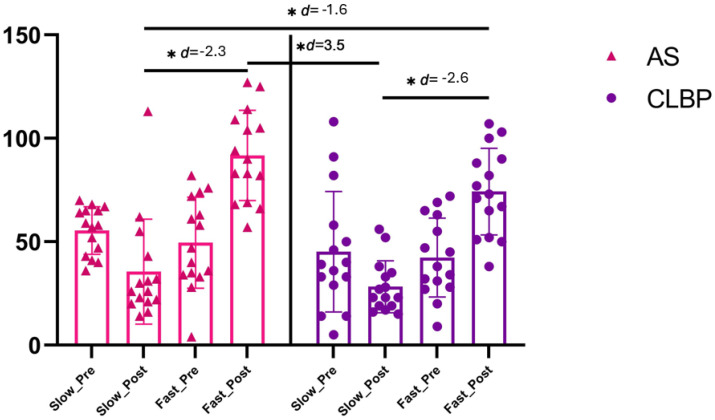
Lumbar flexion velocity between-group results. Abbreviations, AS: asymptomatic subjects; CLBP: chronic low back pain. Note: the asterisk in the figure (*) refers to the ‘*p*’ value associated with that ‘d’ being <0.001.

The ANOVA revealed significant changes in the trunk flexion speed during time (F = 7.9, *p* = 0.007, η^2^ = 0.124) and during group * time interaction (F = 23.69, *p* < 0.001, η^2^ = 0.559). The post hoc analysis revealed significant inter-group differences post-intervention between the AS-S and AS-F groups with a large effect size (mean differences (MD) = −56.1, CI 95% −76.7 to −35.4, *p* < 0.001, d = −2.3), and also between the AS-S and NSCLBP-F groups with a large effect size (MD = −38.6, CI 95% −59.3 to −17.9, *p* < 0.001, d = −1.6). In addition, the post hoc analysis found inter-group differences post-intervention between the NSCLBP-S and AS-F groups with a large effect size (MD = −63.4, CI 95% −84.1 to −42.7, *p* < 0.001, d = −3.5), and also between the NSCLBP-S and NSCLBP-F groups with a large effect size too (MD = −46.0, CI 95% −66.7 to −25.3, *p* < 0.001, d = −2.6) (Figure 3). In addition, the pre- and post-intervention within-group differences were found in all groups (Table S1). Finally, regarding the accuracy analysis, the one-sample *t*-test showed that only the NSCLBP-F group was shown to be accurate with respect to the velocity they observed (MD = −2.7 (−14.3 to 8.8), t(14) = −0.5, *p* = 0.62), while the other groups were not shown to be accurate with respect to the velocity they observed (*p* < 0.05).

### 3.2. Time Up and Go

#### 3.2.1. Total Time Results

**Figure 4:**
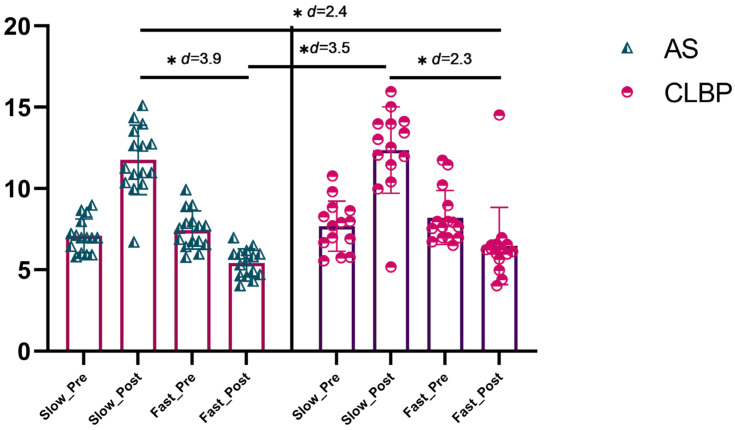
Time Up and Go test between-group results. Abbreviations, AS: asymptomatic subjects; CLBP: chronic low back pain. Note: the asterisk in the figure (*) refers to the ‘*p*’ value associated with that ‘d’ being <0.001.

The ANOVA revealed significant changes in the total time of TUG during time (F = 17.3, *p* < 0.001, η^2^ = 0.240) and also during group * time interaction (F = 31.9, *p* < 0.001, η^2^ = 0.636). The post hoc analysis revealed significant inter-group differences post-intervention between the AS-S and AS-F groups with a large effect size (MD = 6.3, CI 95% 4.2 to 8.4, [36] *p* < 0.001, d = 3.9), and also between the AS-S and NSCLBP-F groups with a large effect size (MD = 5.2, CI 95% 3.1 to 7.3, *p* < 0.001, d = 2.4). In addition, the post hoc analysis found inter-group differences post-intervention between the NSCLBP-S and AS-F groups with a large effect size (MD = 6.9, CI 95% 4.7 to 9.0, *p* < 0.001, d = 3.5), and also between the NSCLBP-S and NSCLBP-F groups with a large effect size too (MD = 5.9, CI 95% 3.7 to 8.0, *p* < 0.001, d = 2.3) (Figure 4). In addition, the pre- and post-intervention within-group differences were found in all groups (Table S2). Finally, regarding the accuracy analysis, the one-sample *t*-test showed that only the AS-F group was shown to be accurate with respect to the velocity they observed (MD = 0.45 (−0.03 to 0.93), t(14) = 2.0, *p* = 0.063), while the other groups were not shown to be accurate with respect to the velocity they observed (*p* < 0.05).

#### 3.2.2. Sit-Up Acceleration Phase Results

**Figure 5:**
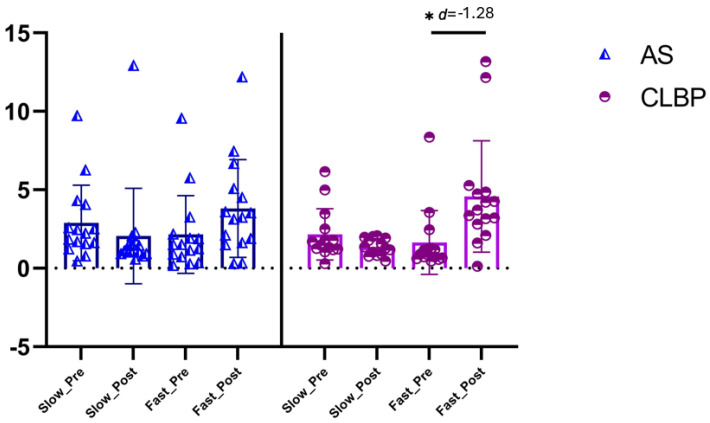
Sit-up acceleration phase between-group results. Abbreviations, AS: asymptomatic subjects; CLBP: chronic low back pain. Note: the asterisk in the figure (*) refers to the ‘*p*’ value associated with that ‘d’ being <0.001.

The ANOVA revealed significant changes in the sit-up acceleration phase during group * time interaction (F = 3.8, *p* = 0.015, η^2^ = 0.172), but not during time (F = 2.3, *p* = 0.12, η^2^ = 0.04). The post hoc analysis revealed significant inter-group differences post-intervention between the NSCLBP-S and NSCLBP-F groups with a large effect size (MD = −3.2, CI 95% −6.1 to −0.3, *p* = 0.022, d = −1.28) (Figure 5).

#### 3.2.3. Sit-Down Acceleration Results

**Figure 6:**
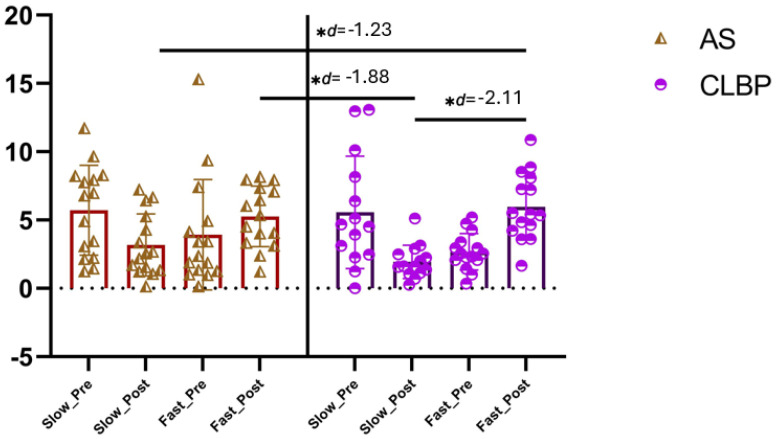
Sit-down acceleration phase between-group results. Abbreviations, AS: asymptomatic subjects; CLBP: chronic low back pain. Note: the asterisk in the figure (*) refers to the ‘*p*’ value associated with that ‘d’ being <0.001.

The ANOVA revealed significant changes in the sit-down acceleration phase during group*time interaction (F = 12.66, *p* < 0.001, η^2^ = 0.408), but not during time (F = 0.7, *p* = 0.40, η^2^ = 0.01). The post hoc analysis revealed significant inter-group differences post-intervention between the AS-S and NSCLBP-F groups with a large effect size (MD = −2.8, CI 95% −4.9 to −0.7, *p* = 0.004, d = −1.23). In addition, the post hoc analysis revealed significant inter-group differences post-intervention between the CLBP-S and AS-F groups with a large effect size (MD = −3.3, CI 95% −5.4 to −1.1, *p* = 0.001, d = −1.88), and also between the NSCLBP-S and NSCLBP-F with a large effect size (MD = −4.0, CI 95% −6.1 to −1.8, *p* < 0.001, d = −2.11) (Figure 6).

## 4. Discussion

The primary objective of this study was to ascertain whether visualizing an action at a specific speed influences the pace of subsequent execution. Regarding lumbar flexion velocity, the findings demonstrated that participants observing the videos at a slow speed, regardless of their NSCLBP status, performed the post-intervention tests significantly slower compared to participants who observed the videos at a high speed. This suggests that the speed of AO altered the outcomes of the lumbar flexion velocity movement.

In the context of the TUG test, generally, we observed analogous outcomes to the primary variable. Participants who observed actions at a slow pace, whether or not they had NSCLBP, executed the test significantly more slowly than those participants who observed the actions at a faster pace. Lastly, concerning the phases of the TUG test, the acceleration variable during the sitting phase behaved in a manner akin to what was previously described, although significant differences during the standing up phase were only found in patients with NSCLBP. The patients observing actions at a slow speed exhibited a significantly lower acceleration when standing up compared to the NSCLBP patients who observed actions at a quick pace. This result was not observed in the Asymptomatic Subjects (AS) group. In summary, it was found that AO training can modify the performance of both functional motor tests according to the observed action’s speed.

The neurophysiological underpinnings of AO training are central to the outcomes observed in this investigation. Fundamentally, AO training stimulates the brain regions implicated in the planning, execution, refinement, and automation of voluntary movements, paralleling the neural engagement during the physical enactment of these actions [40].

The human voluntary movement is planned before the motor cortex sends the signal down the corticospinal pathway to the effector musculature. It is likely that the AO training caused a change in the activity of the areas related to voluntary movement planning, thus influencing the execution of the actual movement following the observation.

The findings from our study, when considered alongside the contributions of Carroll and Bandura (1982, 1985, 1987) and Kim et al. (2017), offer a cognitive perspective, underscoring the significance of visual feedback and motor rehearsal in observational learning and the formation of mental representations of action patterns [41,42,43,44]. These studies suggest that observational learning not only facilitates the acquisition of motor skills, but also refines task-related perception and cognition. The current results extend these notions, indicating that the speed of AO could be a critical factor in modulating these cognitive and perceptual processes. The interaction between AO speed and the perception and execution of movement, highlighted by Allingham et al. (2021) and Székely and Michael (2024), underscores how the speed of the observed movement directly impacts cognition and motor execution. This phenomenon supports the notion that adjusting the speed of AO stimuli could influence the perception of effort and time estimation, which, in turn, could alter the quality of the resultant motor execution [45,46].

Neuroimaging studies in recent years have substantiated these findings, illustrating that AO training not only activates but potentially modifies the neural representation within the ventral premotor cortex [40,47]. In fact, Cantarero et al. showed that AO training is able to elicit changes in the representation of the ventral premotor area [48]. Furthermore, evidence suggests that AO training extends its influence to premotor and supplementary motor areas [49,50], which are intricately involved in the orchestration of voluntary movements. A notable enhancement in corticospinal excitability has also been documented, establishing a positive linkage between cortical reconfiguration and an increase in neuromuscular responsiveness [51,52]. In fact, Ray et al. showed that there is a positive correlation between the change in cortical representation and the level of corticospinal excitability [52]. The research by Moriuchi et al. (2017) and Kitamura et al. (2023) on corticospinal excitability during AO at varying speeds provides a deeper understanding of the neural mechanisms that might explain our observations. The modulation of corticospinal excitability in response to AO speed suggests a pathway through which AO can prime the brain for motor execution, highlighting the significance of adjusting AO speed to replicate movement [53,54].

This ability of people to represent movements is due to the activity of mirror neurons [55]. The foundational mechanism for this capacity to mentally simulate movements lies in the activity of the mirror neurons, identified within crucial motor-related cortical areas, including the premotor cortex, primary somatosensory cortex, posterior parietal cortex, and the supplementary motor area [56]. These neurons provide a neurophysiological substrate for the internal representation of actions, highlighting a profound intersection between observation and motor execution within the human brain.

It is pertinent to mention that Luo et al. (2018) delved into the activation of the mirror neuron system in response to varying AO speed modalities, suggesting that moderate speeds optimize mirror neuron activation [57]. This neurophysiological evidence could partially underpin the findings of our study, providing a theoretical framework to comprehend how variations in AO speed affect motor execution via the modulation of neuronal activation. Temporiti et al. (2020) augment this perspective, underscoring the critical role that specific visual characteristics of AO play in the efficacy of motor rehabilitation [58]. This indicates a need for precision in selecting AO stimuli to maximize motor effects. In our study, evidence suggests that the speed in AO impacts the kinematic outcomes. The concept of stimulus specificity, discussed by Buchanan and Dean (2014), is mirrored in our study, where the speed of AO significantly altered motor execution. These authors emphasize the importance of a consistent movement strategy in AO stimuli to optimize the outcomes in motor function [59].

This could explain the findings found. There is ample literature showing that AO training, both combined with physical exercise and in isolation, is able to cause improvements in maximal strength [60], isometric strength [61], motor control [62], motor learning [63], etc. The rationale used for these improvements is also a neurophysiological argument. However, if we focus on the findings found in this study, there is scientific literature that has found similar results to ours. For example, de la Puente Ranea et al. conducted a study where they evaluated the effect of AO training on the cervical range of motion in patients with chronic neck pain [27]. The patients were assigned to two groups, one with effective videos, where the full range of cervical rotational range of motion was observed, and the other with ineffective videos, where half of the cervical rotational range of motion was observed. After watching the videos, the authors found statistically significant differences in the cervical rotational range of motion, both at the end of the intervention and 10 min after the intervention [27]. That is, the authors found that the video the patients watched influenced the execution of the voluntary movement they performed after the video. In addition, a principle of specificity seems to be observed, since they found no significant differences between the groups when evaluating other cervical movements that they did not observe in the video [27].

In relation to this, similar findings were found by Cuenca-Martínez et al. also in patients with chronic neck pain [62]. The authors conducted a study where they evaluated the OA in isolation (without physical exercise) only in one plane of movement and the effect on the cervical joint position sense in both planes of movement. The authors found that patients improved to a greater degree only in the plane of movement they had previously observed, also finding this principle of specificity of what was observed with respect to what was subsequently performed [62]. This principle of specificity between what was observed and what was executed, added to what was described by Temporiti et al. in 2020 regarding the idea that speed variations could affect motor execution, could partially explain the results obtained in this research on accuracy, according to which only the groups that viewed the videos at a fast speed were accurate [58,62].

However, something that is important to discuss is the finding that the effect of AO training is independent of whether or not there is NSCLBP because the increase or decrease in the speed of execution of the tests evaluated occurred in both groups. At first, we could think that the differences found pre- and post-intervention might not occur, or occur to a lesser extent in patients with NSCLBP compared to AS. We could consider the scenario where pain would have more weight than the effect of the OA in increasing or decreasing the test speed. This is based on the study conducted by La Touche et al., where they found that patients with chronic neck pain showed sympathetic–excitatory response associated with fear of movement when observing actions and images of cervical movements [64]. In addition to this, the study conducted by Perez-Fernandez et al. evaluated the perceived difficulty and fear when observing images of lumbar movements and saw differences between patients with NSCLBP and AS [65]. They found that patients with NSCLBP showed significantly higher levels of fear and perceived difficulty for the observed movements compared to AS. Furthermore, the disability levels predicted a high percentage of variance for both primary variables. This implies that the OA by patients with persistent pain may cause difficulties in planning (and subsequently) executing voluntary movement because it may create fear related to the observed movement. Therefore, we could envisage the scenario discussed above, where patients with NSCLBP would show no differences in the variables analyzed. However, this did not occur. Patients with NSCLBP who observed the slow videos performed the tests more slowly than those who observed the faster videos. It is likely that the key here to explain these findings is that NSCLBP patients did not categorize the observed movements as harmful or dangerous due to low levels of movement-related fear. In fact, future studies could obtain two samples of NSCLBP, one with high levels of movement-related fear and one with low levels of movement-related fear, and see if both populations behave similarly to that found in the present study.

### Study Limitations

Although this study offers valuable insights into the effects of AO speed on motor rehabilitation, it is not without limitations. Firstly, the heterogeneity in AO stimuli, as seen across different studies, suggests that further research is needed to fully understand how various aspects of AO (e.g., speed, complexity, and type of observed action) interact and their optimal settings for rehabilitation purposes.

Moreover, the cross-sectional design of the study limits our ability to infer causality or the long-term effects of AO speed manipulation on motor rehabilitation outcomes. Longitudinal studies are required to determine the sustainability of the observed benefits and whether certain patient populations or specific conditions may respond more favorably to AO interventions.

Although our study included both patients with NSCLBP and asymptomatic individuals, generalizing findings to other conditions or more diverse populations remains to be tested. Future research should explore the efficacy of AO across a broader spectrum of neurological and musculoskeletal conditions, as well as its application in pediatric and elderly populations, to fully leverage its potential in clinical rehabilitation.

Another limitation related to patients may be that the sample is not fully representative due to its size; therefore, it is recommended that future research should include a larger number of subjects.

These studies should also qualitatively record whether the patients in the study have a preconceived idea that low back movements can be considered harmful/dangerous or are capable of causing pain. Finally, another limitation of the study is that it would have been interesting to record measures related to post-intervention pain intensity, to assess whether pain increased when observing lumbar/general movement activities or whether pain intensity might even decrease as NSCLBP patients moved at higher speeds.

The homogeneity of the tasks and AO speed categories used is also a limitation. Future research should explore a wider range of tasks and incremental AO speeds to identify optimal conditions that maximize the benefits of AO training. Additionally, the measurement of motor execution speed using the Baiobit^®^ device may not capture all subtle changes in the quality or speed of motor execution. Incorporating additional measures, such as electromyography or motion analysis, could enrich the dataset and provide a more comprehensive assessment of the effectiveness of AO training.

## 5. Conclusions

The results showed that regardless of presenting with NSCLBP or not, AO training is able to modify the post-observation execution pace in both the lumbar flexion movement and the TUG test. This study shows the capacity that observation has on the planning and execution of voluntary movement, and this should be considered clinically.

## Figures and Tables

**Table 1:** Socio-demographic and self-reported variables.

Variable	AS-S (*n* = 15)	AS-F (*n* = 15)	CLBP-S (*n* = 15)	CLBP-F (*n* = 15)	*p*-Value
Age (years)	41.7 ± 8.3	43.4 ± 6.2	47.9 ± 11.9	48.8 ± 12.2	0.16
BMI (kg/cm^2^)	24.5 ± 5.8	23.7 ± 3.6	25.8 ± 5.9	24.3 ± 3.3	0.67
NPRS (now)	-	-	4.6 ± 1.6	5.0 ± 1.7	-
NPRS (last week)	-	-	6.0 ± 2.2	6.3 ± 1.6	-
Pain frequency (dxm)	-	-	18.6 ± 10.6	23.4 ± 9.3	-
Pain duration (m)	-	-	42.0 ± 46.5	57.4 ± 54.1	-
TSK-11	21.3 ± 4.3	20.5 ± 5.6	28.4 ± 9.2	27.6 ± 8.1	0.004 *
PCS	11.6 ± 8.2	15.0 ± 11.5	21.2 ± 14.5	21.1 ± 15.1	0.11
RMDQ	-	-	5.8 ± 5.5	6.6 ± 4.5	-
CPSS	161.1 ± 12.2	159.3 ± 19.8	138.7 ± 34.3	140.9 ± 26.0	0.02 *
HADS	8.0 ± 3.6	9.8 ± 8.8	11.8 ± 7.3	13.2 ± 7.1	0.21
MIQR-K	22.9 ± 5.1	23.7 ± 4.7	21.4 ± 4.3	22.6 ± 3.6	0.56
MIQR-V	25.1 ± 2.8	25.9 ± 2.5	23.1 ± 4.6	22.5 ± 3.8	0.03 *
Gender					0.39
Female	6 (40)	8 (53.3)	10 (66.7)	10 (66.7)	
Male	9 (60)	7 (46.7)	5 (33.3)	5 (33.3)	
Active working					0.49
Yes	10 (66.7)	10 (66.7)	8 (53.3)	12 (80)	
No	5 (33.3)	5 (33.3)	7 (46.7)	3 (20)	
Medication intake					<0.001 *
Yes	3 (20)	0 (0)	12 (80)	11 (73.3)	
No	12 (80)	15 (100)	3 (20)	4 (26.7)	
Dominant hand					0.277
Right	13 (86.7)	15 (100)	15 (100)	14 (93.3)	
Left	2 (13.3)	0 (0)	0 (0)	1 (6.7)	

* Statistically significant differences; AS: asymptomatic subjects; CLBP: chronic low back pain subjects; F: fast; S: slow; dxm: days per month; m: months; TSK-11: Tampa scale of kinesiophobia; PCS: pain catastrophizing scale; RMDQ: Roland–Morris disability questionnaire; CPSS: chronic pain self-efficacy scale.

## Data Availability

The data presented in this study are available on request from the corresponding author due to privacy restrictions.

## Supplementary Materials

The following supporting information can be downloaded at: https://www.mdpi.com/article/10.3390/brainsci15010031/s1, Table S1: Comparative analysis between groups with respect to the variable trunk flexion speed; Table S2: Comparative analysis between groups with respect to the variable time up and go.
