# Supplementary material for: The Effects of Action Observation Speed on Motor Function in Patients with Chronic Low Back Pain: From Observation to Execution
Source: Brain Sci. 2024 Dec 30;15(1):31. doi: 10.3390/brainsci15010031 (PMC11764160; doi:10.3390/brainsci15010031)
Supplement: Supplementary file 1 [file brainsci-15-00031-s001.zip › brainsci-3390557-supplementary.pdf]

Table S1. Comparative analysis between groups with respect to the variable trunk flexion speed.

Measure	Group	Mean ± SD		MD (95% CI); Effect Size (d)
		Pre	Post	Post-Pre
**Lumbar Flexion Velocity (º/s)**	AS-S	55.4 ± 11.4	35.6 ± 25.3	-19.8* (-33.1 to -6.5); *d*= -1.0
	AS-F	49.5 ± 22.0	91.7 ± 21.8	42.2** (28.9 to 55.4); *d*= 1.9
	CLBP-S	45.2 ± 29.1	28.2 ± 12.5	-16.9* (-30.2 to -3.6); *d*= -0.76
	CLBP-F	42.3 ± 19.0	74.2 ± 20.9	31.9** (18.6 to 45.2); *d*= -1.6
**MD (95% CI); Effect Size (d)**	AS-S vs. AS-F	5.9 (-15.4 to 27.2); *d*= -	-56.1** (-76.8 to -35.4); *d*= -2.3	
	AS-S vs. CLBP-S	10.2 (-11.0 to 31.6); *d*= -	7.3 (-13.3 to 28.0); *d*= -	
	AS-S vs. CLBP-F	13.1 (-8.2 to 34.4); *d*= -	-38.6** (-59.3 to -17.9); *d*= -1.6	
	AS-F vs. CLBP-S	4.3 (-17.0 to 25.6); *d*= -	63.4** (42.7 to 84.1); *d*= 3.5	
	AS-F vs. CLBP-F	7.2 (-14.1 to 28.5); *d*= -	17.4 (-3.2 to 38.1); *d*= -	
	CLBP-S vs. CLBP-F	2.8 (-18.4 to 24.2); *d*= -	-46.0** (-66.7 to -25.3); *d*= -2.6	

*Notes: *p<0.05; **p<0.001; º/s: Grades per second; MD: Mean Differences; CI: Confidence Interval; SD: Standard Deviation; AS: Asymptomatic subjects; CLBP: Chronic low back pain; S: Slow; F: Fast.*

Table S2. Comparative analysis between groups with respect to the variable time up and go.

Measure	Group	Mean ± SD		MD (95% CI); Effect Size (d)
		**Pre**	**Post**	**Post-Pre**
**TUG (seconds)**	AS-S	7.0 ± 1.0	11.7 ± 2.1	4.6** (3.3 to 5.9); *d*= 2.8
	AS-F	7.4 ± 1.1	5.4 ± 0.8	-2.0* (-3.2 to 0.7); *d*= -2.0
	CLBP-S	7.6 ± 1.5	12.3 ± 2.6	4.6** (3.3 to 6.0); *d*= 2.2
	CLBP-F	8.2 ± 1.6	6.4 ± 2.3	-1.7* (-3.0 to -0.4); *d*= -0.9
**MD (95% CI); Effect Size (d)**	AS-S vs. AS-F	-0.3 (-1.7 to 1.0); *d*= -	6.3** (4.2 to 8.4); *d*= 3.9	
	AS-S vs. CLBP-S	-0.6 (-1.9 to 0.8); *d*= -	-0.6 (-2.7 to 1.5); *d*= -	
	AS-S vs. CLBP-F	-1.1 (-2.4 to 0.3); *d*= -	5.2** (3.1 to 7.3); *d*= 2.4	
	AS-F vs. CLBP-S	-0.2 (-1.6 to 1.1); *d*= -	-6.9** (-9.0 to -4.7); *d*= -3.5	
	AS-F vs. CLBP-F	-0.7 (-2.1 to 0.6); *d*= -	-1.0 (-3.1 to 1.0); *d*= -	
	CLBP-S vs. CLBP-F	-0.5 (-1.9 to 0.8); *d*= -	5.9** (3.7 to 8.0); *d*= 2.3	

*Notes: *p<0.05; **p<0.001; MD: Mean Differences; CI: Confidence Interval; SD: Standard Deviation; AS: Asymptomatic subjects; CLBP: Chronic low back pain; S: Slow; F: Fast; TUG: Time Up and Go.*
